# Analysis of Postural Instability in the Upright Position on Narrow Platforms and the Interactions with Postural Constraints

**DOI:** 10.3390/s21113909

**Published:** 2021-06-05

**Authors:** Atsushi Sugama, Akihiko Seo

**Affiliations:** 1 Risk Management Research Group, National Institute of Occupational Safety and Health, Tokyo 204-0024, Japan; 2 Faculty of Systems Design, Tokyo Metropolitan University, Tokyo 191-0065, Japan; aseo@tmu.ac.jp

**Keywords:** postural stability, kinematics, narrow platform, working posture, slips, trips, falls

## Abstract

Background: Loss of balance is a considerable risk factor for workers while using ladders, because they are required to maintain static postural balance on platforms of a restricted size. This study observed center of mass (CoM) and center of pressure (CoP) behaviors and evaluated the effects of the platform depth (anterior–posterior length) and working postures. Methods: Eleven male participants stood on four platforms with depths ranging from 6 to 15 cm and maintained their positions for 60 s while performing or not performing other tasks (object holding, upward viewing, or both simultaneously). The kinematics were analyzed on the sagittal plane based on the inverse pendulum model. Results: The absolute moving range for the CoP–CoM linearly increased with the decreasing platform depth, and the working postures affected the slopes of the linear fits. The relative range of CoP–CoM displacement on narrow platforms was highly correlated with the subjective sense of instability. Conclusions: Monitoring the CoP is effective for a better understanding and evaluation of static postural balance. This study’s findings contribute to improving the design of work equipment through the use of wider platforms that are robust against the effects of working postures.

## 1. Introduction

Loss of balance during work is a serious problem, because workers who lose their balance risk slips, trips, and falls as well as other workplace accidents [1,2]. According to the statistical reports of occupational accidents, falls to lower levels cause 13% of the fatal workplace injuries in the United States [3] and 25% of those in Japan [4]. The related injuries persist as an occupational hazard yet represent a preventable public health problem with a significant societal and economic impact [5,6,7]. Ladders and stepladders are the leading causal agents of occupational falls and are typically the equipment that causes occupational falls from heights. The scenarios and causes are investigated in various countries, such as the United States [8], Sweden [9], Denmark [10], and Japan [11]. In addition, falls often occur for construction workers when working on scaffolds or roofs, which is one of the leading causes of fatal occupational falls [12].

Falls from height are triggered by the loss of balance within the human body or by an unstable environment [13,14]. The postural balance of the human body is maintained through the sensory inputs of the visual, vestibular, and somatosensory systems [15,16,17,18]. Hsiao and Simeonov [19] summarized the elements that affect balance control during roofing work as the following three factors: environmental, task-related, and personal factors. These factors affect the sensory inputs of the worker and can disturb postural stability. For example, the restricted support surface of the equipment is one of the important environmental factors [19]. A support surface is equal to the theoretical base of support for workers. A wider platform is preferred for gaining a wider range of movement for the center of pressure (CoP). Therefore, a narrow platform changes the somatosensory stimulus and increases the postural perturbation while standing or walking on it [17,20,21,22,23]. A wider platform also contributes to reducing the excursion of the vertical projection of the center of mass (CoM) of the body against the base of support [24].

Working postures during tasks also affect the static postural balance [2], which is destabilized by tasks, especially by particular postures such as reaching [25], body movement [26,27], load holding [17,28,29], external loading [30], and mental workload [31,32]. Therefore, these are considered to be task-related factors for human postural stability [19]. However, the interactions between working postures and platform depths have rarely been investigated. Evaluating the postural perturbation is necessary under multiple postural constraints, including working on a restricted surface and the required working postures, to assess the risk of falling in real-world working situations. In addition, if the risk of falling while performing a task depends on the working posture, then monitoring the worker’s postures while performing the tasks is essential.

The hypothesis of the present study is that postural perturbation is affected by interactions between the working posture and the platform depth. The experimental variables are the typical working postures on narrow platforms: object holding (OH), upward viewing (UV), and both conditions simultaneously (OH&UV). To examine the upright postural stability on the narrow platforms, kinetic analysis was performed using the inverse pendulum model with both the CoM and CoP, because postural perturbation due to postural displacements induces body acceleration.

## 2. Materials and Methods

### 2.1. Inverted Pendulum Model and the Extension to Cases on Restricted Platforms

Based on the assumption that the objectives of postural control are the orientation of the CoM and the generation of less perturbation, the position and acceleration of the CoM were used as the objective variables for the postural system. Winter et al. [33] proposed the inverted pendulum model of the body in the sagittal and frontal planes. As is shown in Equation (1), the postural sway on the sagittal plane is described using the anterior–posterior positions of the CoP and CoM as *x*_P_ and *x*_M_, respectively:

(1) $$x_{P}-x_{M}=-\frac{I_{a}{\ddot{x}}_{M}}{Wh}$$

where *I*_a_ is the moment of inertia around the ankle joint and *W* and *h* are the body weight and the height of the CoM above the ankle joint, respectively. For small angular sways, the angular acceleration of the inverted pendulum is nearly equal to the ratio of the horizontal acceleration of the body *ẍ*_M_ divided by the height of the CoM. This equation points to a spring-like behavior between the CoP and CoM (*x*_P_ − *x*_M_) and the acceleration of the body. Therefore, this error signal between the CoP and CoM was used to evaluate the postural perturbation [33].

**Figure 1:**
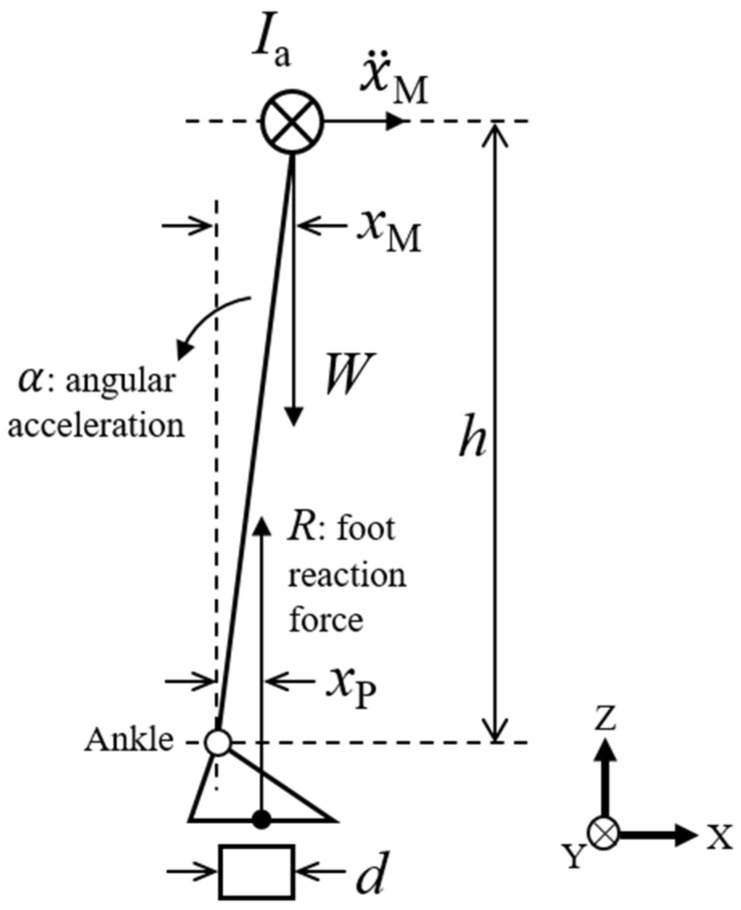
The inverted pendulum model of the human body on a restricted platform in the sagittal plane.

In contrast, while standing on a narrow platform, the CoP position is directly restricted by the platform depth, indirectly resulting in the restriction of the CoM. To evaluate the kinematics while standing on a narrow platform, a model including the platform factor is needed. Figure 1 shows the inverted pendulum model of the body on a restricted platform that is narrower than the human foot’s length. While standing on this narrow platform, the position of the CoP *x*_P_ is limited by the anterior and posterior edges of the platform (*p*_min_ and *p*_max_, respectively). With consideration of Equation (1) and by dividing both sides by the platform depth *d*, which is is defined as *d* = *p*_max_ − *p*_min_, we have

(2) $$\frac{x_{P}-x_{M}}{d}=-\frac{I_{a}{\ddot{x}}_{M}}{Whd}$$

where the relative positions of the CoP and CoM against the platform are respectively defined as *r*_P_ (=*x*_P_/*d*) and *r*_M_ (=*x*_M_/*d*). This equation is expressed as shown in Equation (2):

$$r_{P}-r_{M}=-\frac{I_{a}{\ddot{r}}_{M}}{Wh}$$

The range of *x*_P_ − *x*_M_ is

$$-d<x_{P}-x_{M}<d$$

Here, *d* > 0:

$$-1<r_{P}-r_{M}<1$$

Equation (2) indicates that the difference of the relative positions between the CoP and CoM induces the acceleration of the relative CoM position ${\ddot{r}}_{M}$. Therefore, this study observes the relative position difference (*r*_P_ − *r*_M_) for an index of postural perturbation.

### 2.2. Participants

The study participants were men (sample size = 11, age range = 22–27 years) with little experience in ladder tasks. The sample size was determined based on the results of a similar study [21] using the statistical power calculation software G*Power ver. 3.1.9.2 (Heinrich Heine University Dusseldorf, Düsseldorf, Germany). All participants were healthy and had no medical history of musculoskeletal injuries within the previous 12 months. The means and standard deviations of age, height, and weight were 23.4 ± 1.5 years, 169.3 ± 4.9 cm, and 61.2 ± 7.9 kg, respectively. The vertical heights from the floor to the acromion, trochanter, and tibiale were 138.5 ± 4.1, 86.5 ± 4.1, and 45.5 ± 2.0 cm, respectively. For safety, all participants wore safety harnesses and the same model of safety shoes (MZ010J, Midori Anzen Co., Tokyo, Japan). The participants’ average foot length [34] was 24.7 ± 1.1 cm, and shoe sizes ranged from 24.5 to 27.0 cm. The sole of the safety shoes was made of foamed polyurethane and was non-flat with a bump beneath the arch of the foot. The dynamic slip resistance property of the shoes was certified according to JIS T 8101 F [35]. Participants spent at least 30 min wearing the safety shoes and underwent approximately 10 practice trials before the experiment.

### 2.3. Experimental Apparatus and Procedure

The participants stood on platforms in a comfortable stance with feet shoulder width apart and maintained this position for 60 s. A wooden rectangular parallelepiped platform was placed on a force plate (9286BA, Kistler Group, Winterhur, Switzerland). The height and lateral (left–right) length of the wooden platform were 8 and 48 cm, respectively. The wooden platform’s depth was set at four levels: 6, 8, 10, and 15 cm. The whole-body posture was measured using an optical motion capture system (OptiTrack, Natural Point, Corvallis, OR, USA) with 12 infrared cameras (Flex 3, Natural Point, Corvallis, OR, USA) and its software (Motive 1.8.0, Natural Point, Corvallis, OR, USA). Marker placements of 37 reflective markers, as shown by Yang et al. [36], were used for tracking. The dynamic calibration was achieved by using an L-shaped fixed frame and a moving wand. The calibrated volume measured approximately 3.2 m long, 3.2 m wide, and 2.8 m high. The coordinate system was a right-handed z-up world, and the origin was a point on the platform’s surface.

**Figure 2:**
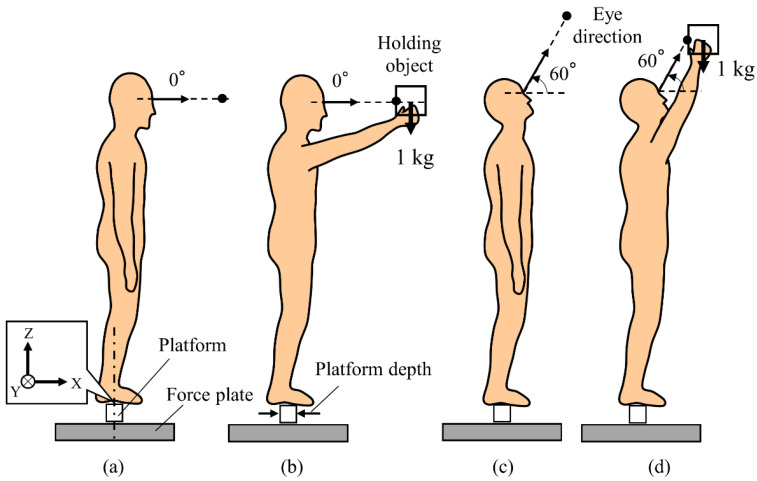
Schematic of experimental conditions: (**a**) standard, (**b**) object holding, (**c**) upward viewing, and (**d**) object holding and upward viewing.

This experiment controlled the platform depth in the anterior–posterior direction and the working postures as the experimental factors. The platform depth was set at four levels: 6, 8, 10, and 15 cm. The depths were determined based on the typical dimensions of ladders, stepladders, and scaffolds, respectively [37,38,39]. The working posture was set at four levels as shown in Figure 2: standard (STD), OH, UV, and OH&UV conditions. In the STD and OH conditions, the participants fixed their vision on a point ahead of them at eye level. Additionally, the OH condition required the participants to hold a 12-cm cubic container (1-kg weight) at eye level using both hands. The UV condition required the participants to view a point located 60° above eye level. In the OH&UV condition, the participants lifted the container upward toward 60° from eye level and viewed the center of the container. Previous studies used various time durations for maintaining a posture of quiet standing, such as 7 s [29], 20 s [40], 30 s [17,41], 60 s [29], and 90 s [42]. This experiment set the duration at 60 s as an intermediate value. Moreover, the holding load was determined assuming that the worker was performing a task involving handling an object in the direction of the ceiling, although the weight used was lighter than the objects handled in previous studies, such as 2.2 kg [30] and 5.2 kg [43].

The subjects performed two trials for each condition. The full combination of experimental factors was 32 trials (4 depths, 4 postures and 2 times). The measurement order was completely randomized for each participant. 

### 2.4. Measurements and Analysis

#### 2.4.1. Kinematic Analysis

**Figure 3:**
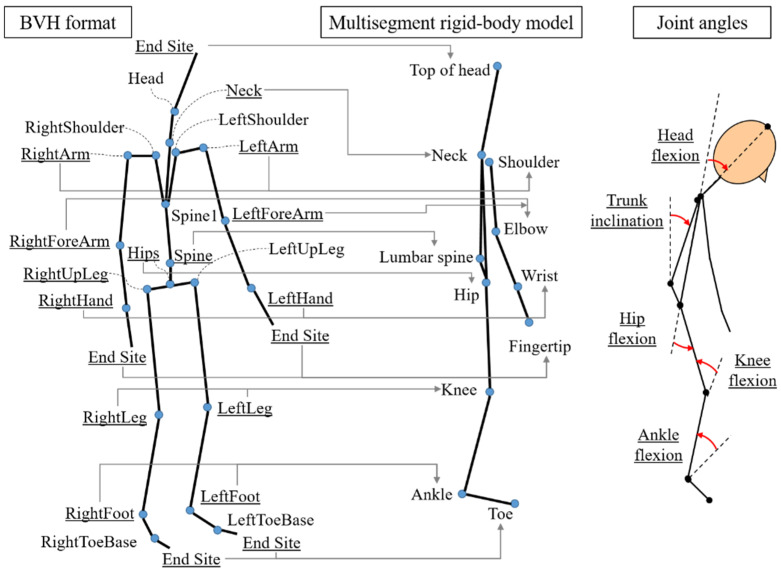
The model of the BVH format and the nodes used for calculating the joint positions and the definitions of the joint angles. The underlined nodes in the BVH model are adopted as the body joints in the multisegment rigid-body model.

The three-dimensional coordinates of the 37 reflective markers placed on the participants’ bodies were recorded at 100 fps. The measured coordinates were converted into Biovision Hierarchy (BVH) format files to calculate the body positions and joint angles. The BVH format files, including the hierarchy of the body skeleton and the rotation angles, were assigned to the whole-body posture of a multisegment rigid body model based on a previous study [44]. A 26-joint skeleton model for the BVH format and the nodes used for calculating the joint positions of a multisegment rigid body model are shown in Figure 3. To project a three-dimensional model onto the sagittal plane, the average position between the right and the left joints was calculated for each joint, excluding the neck, lumbar spine, and hip joints.

The anterior–posterior position of the CoM *x*_M_ was calculated as follows (Equation (3)):

(3) $$x_{M}=\overset{N}{\underset{i=1}{\sum }}\frac{m_{i}x_{i}}{M}$$

where *M* is the body mass, *N* is the number of segments, and *m_i_* and *x_i_* are the mass and anterior–posterior coordinates of the *i*th segments, respectively. The length and mass of each segment were determined as described by Chaffin et al. [45] and Ae et al. [46]. For both the OH and OH&UV conditions, the mass and coordinates of a lifted container were also included in the CoM calculation.

The signals from the force plate were recorded at a sampling frequency of 500 Hz, and they were amplified and used to calculate the anterior–posterior coordinates of the CoP *x*_P_ as follows (Equation (4)):

(4) $$x_{P}=\frac{F_{x}\left(z_{0}+z_{s}\right)+M_{y}}{F_{z}}$$

where *F_x_* and *F_z_* are the measured reaction forces in the X and Z directions, respectively, *M_y_* is the moment of force in the sagittal direction, and *z_0_* and *z*_s_ are the vertical distances from the detection axis of the force plate to its surface and the height of the platform, respectively. These signals were synchronized with the motion capture system and recorded through an analog-to-digital data recording system (PH-703, DKH Co., Tokyo, Japan). The measured signals were low-pass filtered using a second-order Butterworth filter (2-Hz cut-off frequency).

For both the CoM and CoP data, the absolute moving ranges were calculated by subtracting the minimum values from the maximum values. Then, relative moving ranges were calculated by dividing the absolute ranges by the platform depths. In addition, the mean translational velocities for the CoM and CoP were calculated as the sum of *n* time-subtraction data divided by the measurement time *t* as shown in Equation (5):

(5) $$v=\frac{1}{t}\overset{n-1}{\underset{j=1}{\sum }}\sqrt{{\left(x_{j+1}-x_{j}\right)}^{2}}$$

#### 2.4.2. Body Posture

The Euler angle for each joint (°) was obtained from the multisegment model. The mean angles were calculated for the flexion and extension of the neck, hip, knee, and ankle joints, as well as the trunk inclination to a vertical line and the foot inclination to a horizontal line (Figure 3). Then, the stick diagram was plotted as a projection on the sagittal plane.

Based on Equation (5), the mean angular velocity expressed in degrees per second (°/s) was then calculated for the trunk inclination, hip, knee, and ankle using Equation (6):

(6) $$\omega =\frac{1}{t}\overset{n-1}{\underset{j=1}{\sum }}\sqrt{{\left(\theta_{j+1}-\theta_{j}\right)}^{2}}$$

#### 2.4.3. Subjective Sense of Instability

Participants were interviewed after each measurement about their perceptions of total body instability with the following question: “Did you face any difficulty in maintaining balance?” [25]. Each participant assessed their sense of postural instability on a 7-point unipolar Likert scale with the following categories: 1 (feeling no instability), 2, 3, 4 (feeling moderate instability), and 5, 6, and 7 (feeling severe instability).

### 2.5. Statistical Analysis and Function Approximation

Statistical significance of the experimental factors was tested by a repeated measures analysis of variance (ANOVA). The factors of the platform depth (6, 8, 10, or 15 cm) and posture (STD, OH, UV, or OH&UV) were treated as within-subject variables and were tested against their interaction variances (platform depth × posture). Sphericity was checked using the Mauchly sphericity test. When necessary, a correction for sphericity by the Greenhouse–Geisser adjustment was applied. Significant effects were further evaluated by the Bonferroni test. The statistical significance level of all tests was set to 5%. All statistical analyses were carried out using IBM SPSS Statistics 23 (IBM Corp., New York, NY, USA).

The kinematical indices of the CoM and CoP, as well as the subjective sense of instability, were approximated as functions of the platform depth for each working posture condition. The absolute ranges of the CoM, CoP, and CoP–CoM were approximated as linear functions of the platform depth (*y* = *a × d* + *b*) for each working posture. The relative ranges of the CoM, CoP, and CoP–CoM and the subjective sense of instability were approximated as the reciprocal of the platform depth (*y* = *a*/*d* + *b*), while the translational velocities of the CoM and CoP were approximated as functions of the reciprocal of the squared platform depth (*y* = *a*/*d*^2^ + *b*). The coefficients in the approximate equations were calculated by the least squares method.

## 3. Results

### 3.1. Kinematic Indices

**Figure 4:**
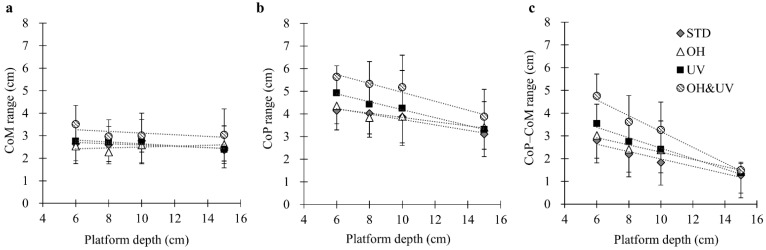
Absolute maximum ranges in the sagittal plane: (**a**) center of mass (CoM), (**b**) center of pressure (CoP), and (**c**) CoP–CoM displacement. Dotted lines show the linear approximate curves for the platform depth (*y* = a*d + *b*). For coefficients of the fitting functions and the significance test of the regression model, see Appendix A.

Figure 4a–c shows the absolute moving ranges for the CoM, CoP, and CoP–CoM, respectively. For the CoM range, the ANOVA showed a main effect of the working posture that significantly increased under the OH&UV condition (*p* < 0.001). The platform depth had no significant effects. Conversely, for the CoP range, the main effects of the platform depth and working posture were significant. The CoP ranges in the 6-cm and 15-cm depth platforms were significantly different from those in the other platform depths. Aside from that, the UV and OH&UV postures increased the CoP range significantly (*p* < 0.001 for all tests). For the CoP–CoM, the main effects of the platform depth and working posture were significant, as well as the interaction. The effects of the working posture were significant, except for the 15-cm depth platform (*p* < 0.001 for all tests). The mean ranges of the CoM were about 2.5 cm for all conditions except for the OH&UV condition, which was about 3.1 cm. The mean ranges of the CoP were wider than that of the CoM in all conditions and ranged from 3.1 to 5.6 cm. The CoP–CoM distance ranged from 2.8 cm to 4.7 cm depending on the working posture under the 6-cm platform, while that was about 1.4 cm for all postures under the 15-cm platform.

**Figure 5:**
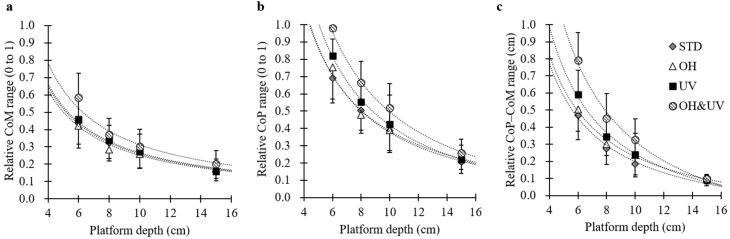
Relative ranges against the platform depth: (**a**) center of mass (CoM), (**b**) center of pressure (CoP), and (**c**) CoP–CoM displacement. Dotted lines show the approximate curves for the reciprocal of the platform depth (*y* = *a*/*d* + *b*). For coefficients of the fitting functions and the significance test of the regression model, see Appendix A.

Figure 5a–c shows the relative ranges against the platform depth for the CoM, CoP, and CoP–CoM, respectively. For all three dependent variables, the relative ranges nonlinearly increased with the decreasing platform depth. The ANOVA showed that the main effects of the platform depth, working posture, and their interaction were significant for all dependent variables. Regarding the interaction between the platform depth and working posture, the effect of the working posture was significant for all platform depths, except for the 15-cm platform. Therefore, the relative ranges for the OH&UV postures became significantly wider with the decreasing platform depth than those for other depths. The relative range for the CoP was near 1.0 under the 6-cm depth × OH&UV posture condition.

**Figure 6:**
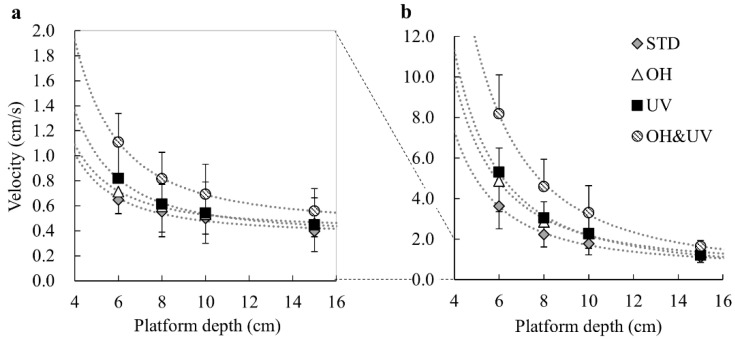
Translational velocity of the (**a**) center of mass and (**b**) center of pressure. Dotted lines show the approximate curves for the negative square of the platform depth (*y* = *a*/*d*^2^ + *b*). For coefficients of the fitting functions and the significance test of the regression model, see Appendix A.

Figure 6a,b shows mean translational velocities of the CoM and CoP. For the CoM velocity, the ANOVA results showed the significant effects of all factors and the interaction between the platform depth and working posture (*p* < 0.001 for all tests). The ANOVA for the CoP velocity showed statistical significance for the platform depth and working posture, as well as the interaction (*p* < 0.001 for all tests). The velocities of the CoM ranged from 0.4 to 1.1 cm/s, whereas that of the CoP ranged from 1.1 to 8.2 cm/s. Both velocities under the OH&UV conditions were significantly higher than in the other conditions.

### 3.2. Body Posture

**Figure 7:**
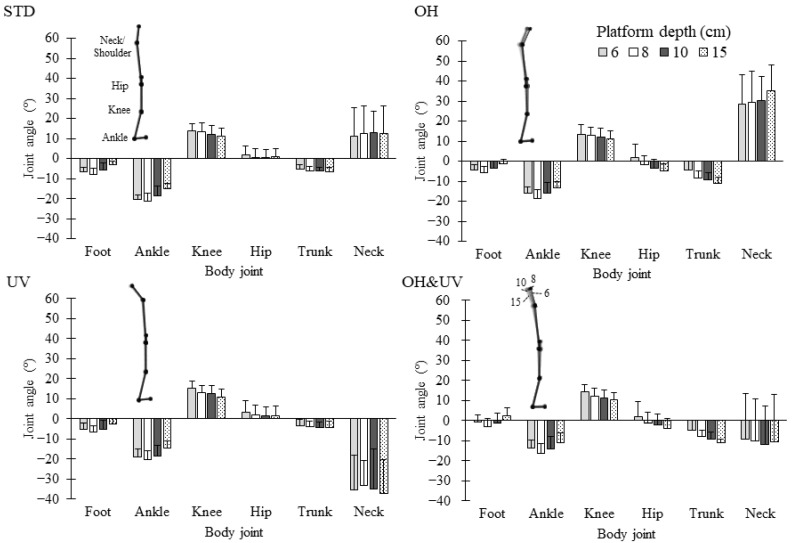
Body joint angle for the trunk and lower limbs and the stick diagram in the sagittal plane. For clarity, the upper limbs are not shown.

Figure 7 presents the mean joint angles for flexion and extension and the stick diagrams projected on the sagittal plane. The ANOVA for the neck angle showed the significant effect of the working posture, whereas the platform depth had no significant effect. The mean neck angle for the STD condition was 11° of flexion. The OH posture increased the angle to 30°, whereas the UV posture decreased the angle to −35°. As a result, the mean angle under the OH&UV condition was −10°. The trunk inclination angles had negative values (backward tilt) for all conditions. The OH posture significantly increased the backward tilt. In contrast, the UV posture made the angle close to zero. The hip angles were significantly affected by an interaction of the platform depth and the working posture (*p* < 0.001). The hip angle had negative (extension) values for the OH and OH&UV postures versus positive (flexion) values for the STD and UV postures, although the differences among the working postures were less than 10°. The ANOVA for the knee joint angle showed only a significant effect for the platform depth (*p* < 0.001). Significant differences were also noted among all depths, and the knee angle increased from 10.9° up to 15.1° with narrower depths. The ANOVA for the ankle joint angle showed the significant effects of the platform depth and the working posture (*p* < 0.001 for all tests). The ankle angle was negative (dorsiflexion) under all conditions. When comparing the platform depth levels, the 8-cm depth platform had the maximum angular displacement (19° of dorsiflexion) from the neutral position. Compared with the dorsiflexion angle of 19° for the STD posture, the OH posture decreased the angle to 16°, and the OH&UV posture decreased the angle to 13°.

**Figure 8:**
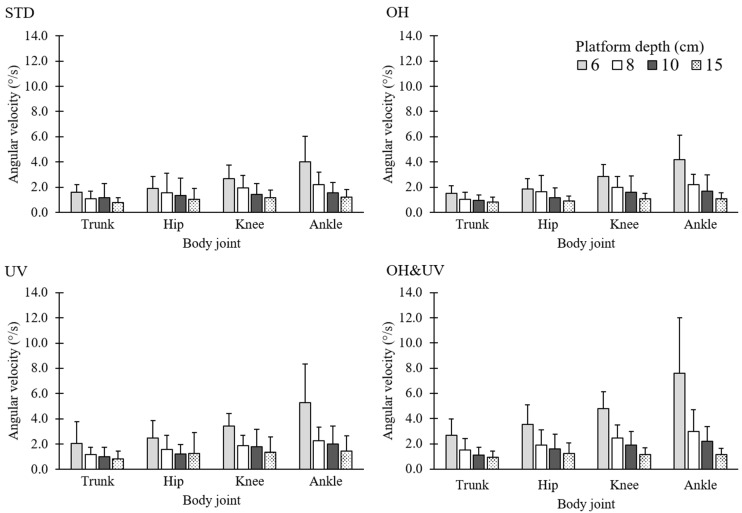
Angular velocity for the trunk, hip, knee, and ankle.

Figure 8 shows the angular velocities of the trunk inclination, hip, knee, and ankle joints. The ANOVA for the trunk and hip angular velocities showed similar significant effects for the platform depth and working posture as well as their interactions (*p* < 0.001 for all tests). For both the trunk and hip joints, the angular velocity significantly increased with the 6-cm platform depth and decreased with the 15-cm depth, although the increase was noted only for the OH&UV posture with the 6-cm depth. The maximum angular velocities for the trunk and hip were 2.7°/s and 3.5°/s, respectively, under the conditions of a 6-cm platform depth and the OH&UV posture. The ANOVA for the knee showed significant effects from the platform depth and working posture (*p* < 0.001 for all tests) as well as the interaction (*p* < 0.001). The multiple comparison test indicated significant differences among all levels of the platform depth. Additionally, the angular velocity increased for the OH&UV posture only with the 6-cm platform depth. The ANOVA for the ankle showed significant effects from the platform depth and working posture (both *p* < 0.001) as well as the interaction (*p* < 0.001). The effects of the OH&UV posture were significant for the 6-cm platform depth. The maximum angular velocity for the ankle was 7.6°/s under the conditions of the 6-cm platform depth and the OH&UV posture.

### 3.3. Subjective Sense of Instability

**Figure 9:**
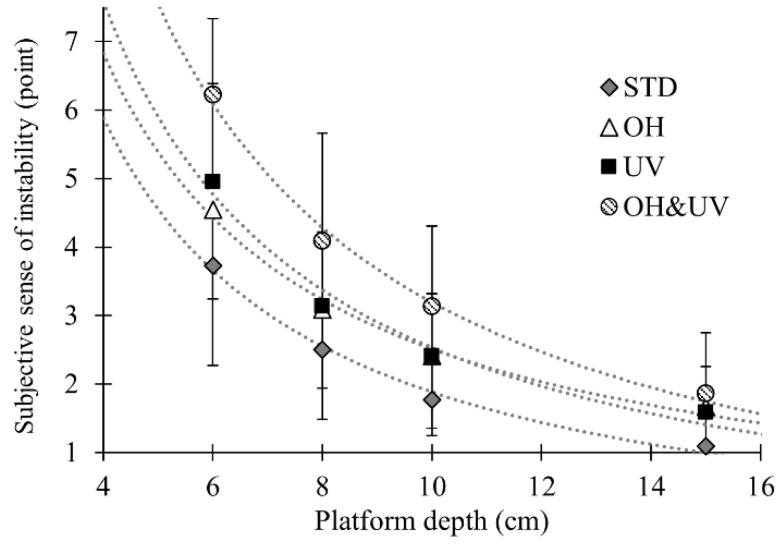
Subjective sense of instability as a function of the platform depth. Dotted lines show the approximate curves for the reciprocal of the platform depth (*y* = *a*/*d* + *b*). For coefficients of the fitting functions and the significance test of the regression model, see Appendix A.

Figure 9 represents the subjective sense of instability. The ANOVA showed the significant effects of the platform depth and working posture, as well as their interaction (*p* < 0.001 for all tests). The multiple comparisons showed significant differences between all levels of the sense of instability. The effects of the OH&UV posture were significant for all platform depths except the 15-cm depth.

**Figure 10:**
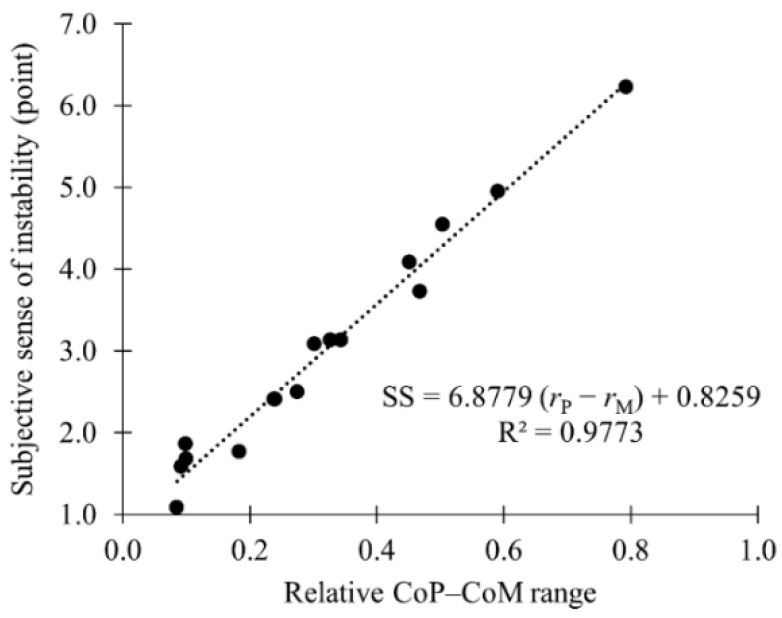
Relationship between the subjective sense of instability (SS) and the relative range of the center of mass (CoM) to the center of pressure (CoP) distance (*r*_P_ − *r*_M_).

Figure 10 shows the relationship between the subjective sense of instability and the relative range of the CoP–CoM distance (*r*_P_ − *r*_M_). The plotted data consist of the mean value of each condition. The correlation analysis showed a high linear correlation between the two indices (*r* = 0.988).

## 4. Discussion

### 4.1. Movements of CoM and CoP on Narrow Platforms

The platform depth affected only the absolute CoP range and not the absolute CoM range. Therefore, the CoP–CoM displacement was mainly increased by the widening of the CoP ranges. These phenomena were considered to depend on the decreased contact area between the soles of the feet and the platform. Previous studies suggested that the human postural control system acquires information about the body’s position and movement through the visual, vestibular, and somatosensory systems and organizes this information in the central nervous system [24]. The weight of the input information from each system is varied by the surrounding environment, and the loss or decline of input information is associated with an inaccuracy of postural control [16,47]. For example, inputs from the vestibular system take priority over other systems when a person is on an inclined floor, whereas people attach more importance to inputs from the somatosensory system, such as muscles, tendons, and joint receptors, when on flat and deep platforms [48,49]. Therefore, the probable insufficiency of somatosensory input from the soles of the feet contributed to the increased postural perturbation with the decreasing platform depth.

The relative ranges of the CoM and CoP, dividing the absolute ranges by the platform depths, covered about 40–70% and about 70–100% for the 6-cm platform depth, respectively. Because these indices were proportional to the reciprocal of the platform depth, the values increased nonlinearly with the narrowing depth. The reason for these trends is considered to be the kinematic need. When the body is slightly inclined, the CoP is moved in the same direction as the CoM and then surpasses the CoM to generate the horizontal acceleration in the opposite direction. Therefore, for the control system of static postural balance, the dependent variable is the CoM position, while the independent variable is the CoP position. However, on narrow platforms, the controllable ranges of the CoP and CoP–CoM distance are theoretically restricted to the platform depth in the anterior–posterior direction. Therefore, participants needed to move their CoPs faster and ensure certain CoP–CoM distances. As a result, the CoP velocity was considered to be nonlinearly affected by the platform depth.

### 4.2. Postural Adjustments and Control

As for the effects of the working posture, the moving range and velocity of the CoP increased in the order of STD < UV < OH < OH&UV. For the OH posture, the masses of the handled container and elevated upper limbs transfered the whole-body CoM forward. Therefore, participants needed to adjust the CoM position over the platform’s center by inclining the trunk backward and rotating the head forward. Next, for the UV posture, backward rotation of the neck was required to gaze upward. The neck rotation may worsen the accuracy of the input from the vestibular system, and gazing at a fixed point upward may make it difficult to control the static postural balance based on visual information. For both the OH and UV postures, constraints on the static postural balance were generated by postural adjustments during task performance. Consequently, OH&UV postures required complex postural changes. The neck angles during the OH&UV postures showed similar trends with the UV postures, whereas the trunk and hip angles were similar to the OH postures. These results suggest that different methods of postural control are adopted for comprehensive adjustments, depending on the importance and constraints of the ongoing tasks and the local environment.

The angular velocities increased for lower joints and narrower platforms. These results indicate that postural control in the sagittal plane on narrow platforms was primarily realized by the ankle joint’s motion. These phenomena agree with the findings of previous studies. Horak and Nasher [50] suggested two strategies for postural control: the ankle strategy and the hip strategy. The ankle strategy consists of muscular activities for the flexion and extension of ankle joints as simulated inverted pendulums [50]. The hip strategy involves quick hip motion generating the inertial force to move the CoM and regain balance, and this strategy is also used when individuals stand on narrow steps. In this study, it was considered that the participants adopted the ankle strategy for steady postural control and then used the hip strategy as needed for acute or further perturbations.

### 4.3. Sensing and Evaluation of Postural Stability

Pai et al. [51] noted that both the position and velocity of the CoM must be considered concurrently to evaluate postural stability. In this study, the effects of task-related factors on postural perturbation increased with the decreasing platform depth. Additionally, no significant difference was observed between the kinematic indices of the CoP when the depth of the platform was 15 cm. Therefore, using equipment with platforms of 15 cm or deeper can contribute to minimizing the effects of task-related factors on postural perturbation, which is important for the safe performance of tasks while standing on equipment at a height. The subjective sense of instability observed in this study was also proportional to the relative range of CoP–CoM displacement. The reason for this may be that the subjective sense of instability is associated with body acceleration due to the postural perturbations described previously.

The degree of environmental severity for maintaining the static postural balance dominantly increased the CoP movement and not the CoM’s, because the main independent variable for the postural control system was the CoP position. Therefore, monitoring the CoP is effective for a better understanding and evaluation of static postural balance. In contrast, monitoring the CoM using such tools as an accelerometer contributes to evaluating the outputs of the balance system. Moreover, using wearable technologies such as inertial sensors, surface electromyography sensors, and pressure sensors will contribute to the evaluation and detection of falls [13,52]. A more precise estimation of foot reaction forces and the CoP using wearable sensors will contribute to monitoring the relative range of CoP–CoM and a better understanding of the physiology and pathophysiology of balance [53]. For example, studies using wearable sensors for balance evaluation in neurological disorders show high correlations between inertial and CoP measures for cerebellar ataxia and neuropathies [13].

### 4.4. Application to Work Environments and the Limitations

The design requirements for the step depth of ladders are often referred to in the bills for occupational safety and health or industrial standards. For example, in the United States, OSHA standard 1910.23(e)(2)(i) requires a depth of no less than 7 inches (18 cm) for mobile ladder stands and mobile ladder stand platforms. The European standards in the BS EN-131 series define a standing surface less than 80 mm in depth a “rung“ and a surface greater than 80 mm in depth a “step“. The results of the present study show that deeper steps improved the static postural balance, thus reducing perturbation due to interactions between the working postures and platform depth. Therefore, equipment with a working platform deeper than at least 15 cm is recommended for tasks performed while standing on equipment to minimize the risk of loss of balance.

The limitations of this study include the limited sample size and the effects of factors such as sex, age, mental workload, and pathology. Studies including larger sample sizes and female participants may evaluate more accurately the postural perturbation and the subjective sense of postural instability. Moreover, older adult participants may be susceptible to the narrowness of platforms and task-related factors because of declining functionality of the musculoskeletal system and the sensory organs. Additionally, some individuals with neurological disorders lead to impaired sensorimotor function and postural responses, abnormal central proprioceptive-motor integration or cerebellar function, or impaired coordination of movements [13]. In these individuals, complex postural constraints requiring elaborate postural orientation and coordination, such as the OH&UV posture, should be avoided, because the interactions between the step depth and postures potentially worsen the postural perturbation. Therefore, limiting these postures and promoting the use of equipment with deeper steps may contribute to the safer design of equipment to minimize various factors that increase postural perturbation.

Additionally, psychological and emotional factors may affect the static postural balance. In the real field of construction, workers standing at higher places for ladder tasks need to maintain their static postural balance under severe mental workloads. DiDomenico and Nussbaum showed that mental workloads have interactive effects with postural demands [31]. Therefore, ladder tasks in higher places potentially induce postural perturbation further than that observed in the present study. To examine these effects, experiments conducted with the visual effects of a high platform should be undertaken to reflect real-world situations in the field of construction. To safely simulate elevated workplaces, the use of virtual reality technology is effective, as was shown in previous studies [16,54].

## 5. Conclusions

This study observed the CoM and CoP during static standing on narrow platforms to evaluate the postural perturbation and effects of working postures. The absolute ranges of the CoP and CoP–CoM displacement increased linearly with the narrowing of the platform depth, whereas that of the CoM was constant with the platform depth. Additionally, the working postures of OH, UV, or both (OH&UV) affected the slopes of the linear fitting function. As a result, the relative moving range of the CoP and CoP–CoM, dividing the absolute ranges by the platform depths, changed nonlinearly with the narrowing platform depth, thus showing interactions with working postures. The translational velocity of the CoP also increased nonlinearly for restricted platform depths, which is considered to be due to the kinematic constraints, ensuring CoP–CoM displacement on narrow platforms. These results indicate that monitoring the CoP is effective for a better understanding and evaluation of static postural balance. In addition, these findings contribute to the evidence for improving the design of occupational equipment such as ladders to use wider platforms that are robust against the effects of working postures, which may thus decrease the risk of falling during tasks.

The responses of the postural control system against restricted platform depths were observed to be increases of the angular velocities for ankle and knee joint motions. The effects of the working posture were mainly observed as the differences of the mean joint angles. The compensations for the weights of the handled object and elevated arms for the OH postures and the backward rotation of the neck for the UV postures were considered to be major causes of the postural changes. The case of simultaneous OH and UV (OH&UV) required different types of parallel postural adjustments and generated larger postural perturbation. The subjective sense of postural instability was highly correlated to the relative range of the CoP–CoM displacement against the platform depth. The postural balance model extended for postures on restricted platforms showed that the relative range was considered to be reasonable as the evaluation index for postural perturbation. Therefore, use of the subjective sense of instability seems to be effective for monitoring the static postural balance with no use of measuring devices.

## Data Availability

The data presented in this study are available on request from the corresponding author. The data are not publicly available due to lack of consent for sharing individual data.

## Appendix A. Supplemental Data for the Approximate Function

Table A1 shows the ANOVA results. Table A2 gives the coefficients of approximate functions for kinematical indices and the subjective sense of instability.

**Table A1:** Results of repeated measures ANOVA for the kinematical indices and the subjective sense of instability (** *p* < 0.01. *: *p* < 0.05; n.s.: not significant).

	**Absolute Range of CoM**			**Absolute Range of CoP**			**Absolute Range of CoP–CoM**		
**Factor: DoF**	**F Value**	**Significance**	**Multiple** **Comparison**	**F Value**	**Significance**	**Multiple** **Comparison**	**F Value**	**Significance**	**Multiple** **Comparison**
Platform depth: F (3, 30)	1.30	n.s.		20.96	**	6 cm > 10, 15 cm; 15 cm < 8, 10 cm	73.50	**	** for all pairs except for an 8–10 cm pair
Working posture: F (3, 30)	10.47	**	OH&UV > STD, OH, and UV	29.54	**	STD < UV, OH&UV; OH&UV > OH and UV	29.34	**	STD < UV, OH&UV; OH&UV > STD, OH, and UV
Platform depth × Working posture: F (9, 90)	1.20	n.s.		1.02	n.s.		4.33	** under the adjusted DoF: F (3.88, 38.75)	Effects of working posture: n.s. for 15-cm depth, and ** for other depths
Platform depth: F (3, 30)	191.06	** under the adjusted DoF: F (1.78, 17.76)	** for all pairs	367.00	**	** for all pairs	278.85	**	** for all pairs
Working posture: F (3, 30)	11.29	**	OH&UV > STD, OH, and UV	36.25	**	** for all pairs except for an STD–OH pair	31.46	**	STD < UV; OH&UV > STD, OH, and UV
Platform depth × Working posture: F (9, 90)	2.52	**	Effects of working posture: n.s. for 10- and 15-cm depths, and ** for 6- and 8-cm depths	3.04	**	Effects of working posture: n.s. for 15-cm depth, and ** for other depths	6.98	** under the adjusted DoF: F (3.88, 38.75)	Effects of working posture: n.s. for 15-cm depth, and ** for other depths
	**Translational Velocity of CoM**			**Translational Velocity of CoP**			**Subjective Sense of Instability**		
**Factor: DoF**	**F Value**	**Significance**	**Multiple** **Comparison**	**F Value**	**Significance**	**Multiple Comparison**	**F Value**	**Significance**	**Multiple** **Comparison**
Platform depth: F (3, 30)	64.95	** under the adjusted DoF: F (1.77, 17.68)	** for all pairs	110.35	**	** for all pairs	74.66	**	** for all pairs
Working posture: F (3, 30)	44.14	**	STD < UV; OH&UV > STD, OH, and UV	76.98	**	** for all pairs except for an OH–UV pair	34.14	**	** for all pairs except for an OH–UV pair
Platform depth × Working posture: F (9, 90)	3.32	**	Effects of working posture: in 15-cm depth, ** only for an STD-OH&UV pair	15.74	**	Effects of working posture: n.s. for 15-cm depth, and ** for other depths	4.32	**	Effects of working posture: * for 15-cm depth, and ** for other depths

**Table A2:** Coefficients of fitting functions for kinematical indices and the significance test of regression model (** *p* < 0.01; n.s.: not significant). The models of the fitting functions were the linear for the platform depth (*y* = *a × d* + *b*) for the absolute range, the reciprocal of the platform depth (*y* = *a*/*d* + *b*) for the relative range and the subjective sense of instability, and the negative square of the platform depth (*y* = *a*/*d*^2^ + *b*) for the translational velocity.

	**Absolute Range of CoM**				**Absolute Range of CoP**				**Absolute Range of CoP–CoM**			
	**STD**	**OH**	**UV**	**OH&UV**	**STD**	**OH**	**UV**	**OH&UV**	**STD**	**OH**	**UV**	**OH&UV**
*a*	−0.029	−0.020	−0.042	−0.037	−0.119	−0.067	−0.174	−0.197	−0.161	−0.160	−0.230	−0.347
*b*	2.875	2.306	3.053	3.482	4.949	4.203	5.925	6.930	3.601	3.882	4.759	6.667
Adjusted R^2^ for raw data	0.001	0.009	0.010	0.007	0.133	0.023	0.191	0.260	0.359	0.312	0.415	0.578
Significance of regression model (F value)	n.s. (1.12)	n.s. (0.74)	n.s. (1.91)	n.s. (1.65)	** (13.0)	n.s. (1.93)	** (21.53)	** (30.54)	** (49.66)	** (40.42)	** (51.94)	** (117.49)
*a*	2.746	2.407	2.966	3.793	4.822	4.607	5.956	6.737	3.842	3.969	4.964	6.850
*b*	−0.015	−0.009	−0.032	−0.070	−0.106	−0.081	−0.177	−0.177	−0.187	−0.169	−0.252	−0.368
Adjusted R^2^ for raw data	0.485	0.549	0.535	0.653	0.689	0.635	0.765	0.828	0.689	0.704	0.724	0.799
Significance of regression model (F value)	** (82.92)	** (106.93)	** (101.15)	** (164.80)	** (191.72)	** (152.39)	** (284.55)	** (405.64)	** (194.77)	** (208.01)	** (226.78)	** (338.92)
	**Translational Velocity of CoM**				**Translational Velocity of CoP**				**Subjective Sense of Instability**			
	**STD**	**OH**	**UV**	**OH&UV**	**STD**	**OH**	**UV**	**OH&UV**	**STD**	**OH**	**UV**	**OH&UV**
*a*	2.466	10.669	15.784	10.669	106.85	145.85	173.58	280.93	26.65	28.75	33.65	43.51
*b*	0.241	0.421	0.376	0.421	0.637	0.725	0.450	0.373	−0.781	−0.362	−0.833	−1.156
Adjusted R^2^ for raw data	0.212	0.237	0.261	0.237	0.637	0.658	0.760	0.755	0.524	0.495	0.573	0.637
Significance of regression model (F value)	** (24.37)	** (27.99)	** (31.74)	** (27.99)	** (151.60)	** (166.10)	** (277.12)	** (260.65)	** (96.57)	** (86.19)	** (117.51)	** (153.42)
